# Primary Astrocytic Tumours and Paired Recurrences have Similar Biological Features in IDH1, TP53 and TERTp Mutation and MGMT, ATRX Loss

**DOI:** 10.1038/s41598-017-13272-9

**Published:** 2017-10-12

**Authors:** Xia Li, Jie Wei, Yixiong Liu, Peifeng Li, Linni Fan, Yingmei Wang, Mingyang Li, Danhui Zhao, Zhou Yu, Jing Ye, Ying Guo, Qingguo Yan, Shuangping Guo, Zhe Wang

**Affiliations:** State Key Laboratory of Cancer Biology, Department of Pathology, Xijing Hospital; and School of Basic Medicine, The Fourth Military Medical University, Xi’an, Shaan Xi Province China

## Abstract

Astrocytic tumours are the most common type of primary malignant brain tumour. Most astrocytic tumours will recur at some point after surgery. Currently, the combination of radiotherapy and chemotherapy does not prevent the recurrence of astrocytic tumours. In this study, we investigated the consistency in isocitrate dehydrogenase 1 (*IDH1*), tumour protein p53 (*TP53*) and telomerase reverse transcriptase promoter (*TERTp*) mutations during astrocytic tumour recurrence. We also evaluated the protein loss of O-6-methylguanine-DNA methyltransferase (MGMT) and alpha-thalassemia/mental retardation, X-linked (ATRX) during disease recurrence. We then determined the prognostic significance of these findings in terms of progression-free survival (PFS) using Kaplan-Meier analysis and Cox regression models. Our results showed that in most cases, *IDH1*, *TP53* and *TERTp* mutation status and MGMT and ATRX protein expression levels were stable during recurrence, which may indicate that these alterations occurred early in astrocytic tumour development. Furthermore, in *IDH1* wild type group, the patients who were negative for MGMT and had a low Ki67 index showed a longer PFS. Therefore, we suggest that *IDH1* mutation combined with MGMT expression level and Ki67 index might be an effective biomarker panel for evaluating the PFS of patients with astrocytic tumours.

## Introduction

Astrocytic tumours are the most common primary malignant tumours in the central nervous system^1^. The World Health Organization (WHO) classification system divides astrocytic tumours into four levels (I, II, III and IV) according to biological behaviour^2^. The majority of astrocytic tumours, except grade I astrocytomas, will recur at some point after surgery, and some recurrent cases evolve from a lower grade tumour (grade II or III) to a higher grade tumour (grade III or IV)^3,4^. At present, the prognosis of high-grade tumours is poor; for instance, the 5-year survival rate of patients with glioblastoma is less than 5%, and the average survival time is only 14 months^5^. To date, there is no effective way to prevent the recurrence of astrocytic tumours and no effective treatment for recurrent disease^6^. Recurrence is one of the most important reasons for the poor outcomes associated with astrocytic tumours.

In the last few years, considerable advances have been made in the sequencing of astrocytic tumours, which led to the discovery of key genetic alterations in gliomas, including mutations in the isocitrate dehydrogenase 1/2 (*IDH1/2*), homologue of Drosophila capicua (*CIC*), far-upstream binding protein 1 (*FUBP1*), and alpha-thalassemia/mental retardation, X-linked (*ATRX*) genes and in the *TERT* promoter (*TERTp*) region^7–10^. These biological molecules are strongly correlated with the traditional tumour classification and prognosis^10–12^. In 2012, Jiao *et al*.^12^ introduced a new way to classify gliomas and evaluate prognosis by integrating *ATRX*, *CIC*, *FUBP1* and *IDH1* mutations into the classification. The resulting three categories were as follows: I-A (*IDH1/ATRX* mutation), I-CF (*IDH1/CIC/FUBP1* mutation), and I-X gliomas (not I-A or I-CF). The median overall survival of patients with I-CF gliomas (96 months) was longer compared to that of patients with I-A gliomas (51 months) or I-X gliomas (13 months)^12^. In the following years, several studies explored the classification of gliomas by integrating *TERTp*, *IDH1/2*, *TP53*, and *ATRX* mutations and 1p/19q co-deletions^13–18^. Therefore, the above biomarkers became important auxiliary tools for the diagnosis, prognosis and treatment of gliomas. At present, the WHO classification system includes these molecular parameters, in addition to histology, to distinguish among the different types of glioma^18^, opening the way to the era of molecular diagnosis of gliomas.

In terms of the abovementioned molecular parameters, key mutations in astrocytic tumours include classic mutations in the *IDH1* and *TP53* genes and mutations affecting telomere length in the *ATRX* gene and *TERT* promoter region. MGMT promoter hyper-methylation is another important epigenetic marker that might result in gene expression silencing and further impair the ability to repair DNA. Patients with astrocytic tumours might benefit from temozolomide because of the presence of MGMT gene silencing^19^. Previous studies on these gene alterations, especially those of *TERTp* and the *ATRX* gene, have mostly focused on primary tumours, with little investigation of paired recurrent tumours. Furthermore, studies on the function of *IDH1*, *TP53*, *ATRX* and *TERTp* as prognostic biomarkers for the progression-free survival (PFS) of patients with astrocytic tumours are controversial^11,19–29^. Therefore, the aim of this study was to evaluate the consistency of key genetic alterations in *IDH1*, *TP53* and *TERTp* mutation status and ATRX and MGMT protein expression level in the course of astrocytic tumour recurrence; furthermore, the prognostic significance of these findings for PFS was discussed.

## Results

### Frequency of *IDH1*, *TP53*, and *TERTp* mutation and loss of MGMT and ATRX in the evolution of astrocytic tumours

We compared sequence alterations in *IDH1*, *TP53* and *TERTp* and changes in the protein level of MGMT and ATRX in 47 primary astrocytic tumours and 48 corresponding recurrences. In the primary tumours, the frequencies of *IDH1* and *TP53* mutations were higher in astrocytoma (A) and anaplastic astrocytoma (AA) than in pGBM (primary glioblastoma). In contrast, *TERTp* mutations were rarer in A and AA than in pGBM. In recurrent tumours, the frequencies of *IDH1* and *TP53* mutations were higher in A, AA and secondary glioblastoma (sGBM) than in rGBM (recurrence of pGBM), and the frequency of *TERTp* mutations was lower in A, AA and sGBM than in rGBM. Regarding protein loss (negative immunohistochemical staining results), ATRX loss in primary tumours was more common in A and AA than in pGBM, whereas MGMT loss was not significantly different among all tumour grades. In recurrent tumours, ATRX loss was more common in A, AA and sGBM than in rGBM; MGMT loss was not significantly different in A, AA, sGBM and rGBM. The *TERTp* mutation pattern showed a remarkable inverse correlation with *IDH1* and *TP53* mutations and ATRX protein loss. The specific genetic mutation frequencies and protein loss frequencies in primary and recurrent tumours are shown in Table 1.

**Table 1:** Frequency of *IDH1*, *TP53* and *TERTp* mutations and MGMT and ATRX loss in the evolution of astrocytic tumours.

Histology		total	*IDH1*R132		*TP53*		*TERT*p		MGMT		*ATRX*	
			mutation	frequency	mutation	frequency	mutation	frequency	loss	frequency	loss	frequency
Prim	A	9	7	77.78%	5	55.56%	1	11.11%	5	55.56%	6	66.67%
	AA	15	11	73.33%	10	66.67%	2	13.33%	11	73.33%	6	40.00%
	pGBM	23	3	13.04%	2	8.70%	12	52.17%	15	65.21%	3	13.04%
	total	47	21	44.68%	17	36.17%	15	31.91%	31	65.96%	15	31.91%
Rec	A	3	3	100%	1	33.33%	0	0.00%	2	66.67%	2	66.67%
	AA	9	7	77.78%	7	77.78%	1	11.11%	5	55.56%	4	44.44%
	sGBM	12	8	66.67%	7	58.33%	1	8.33%	10	83.33%	5	41.67%
	rGBM_1_	23	3	13.04%	2	8.70%	14	60.87%	14	60.87%	3	13.04%
	rGBM_2_	1	1	100%	0	0	0	0	0	0	0	0
	total	48	22	45.83%	17	35.42%	16	33.33%	31	64.58%	14	29.17%

Prim, primary; Rec, recurrence; A, astrocytoma; AA, anaplastic astrocytoma; pGBM, primary glioblastoma; sGBM, secondary glioblastoma; rGBM_1_, the first recurrence of pGBM; rGBM_2_, the second recurrence of pGBM.

### *IDH1*, *TP53* and *TERTp* mutation sites in astrocytic tumours

**Figure 1:**
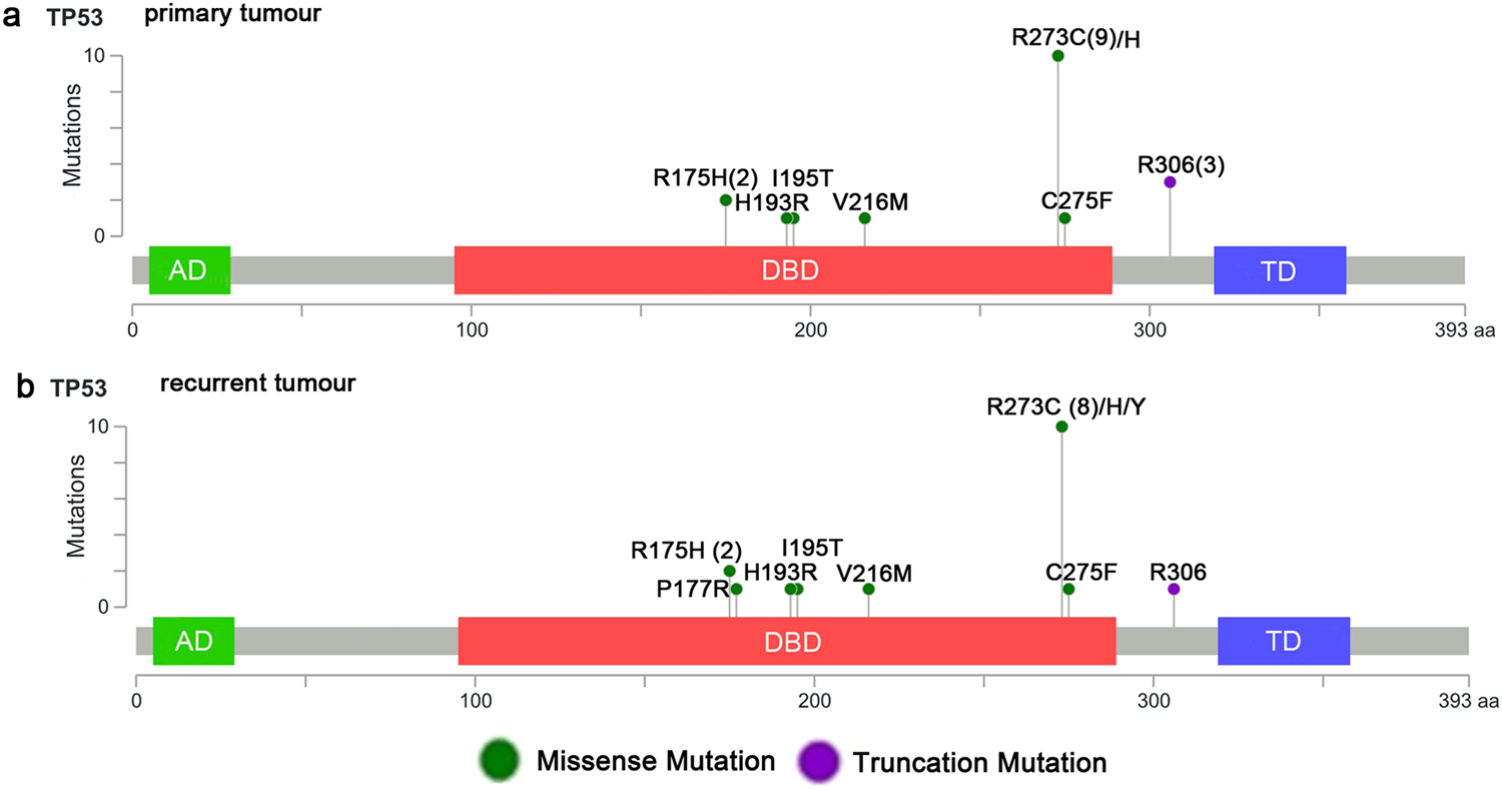
Mapview of *TP53* gene mutations in 47 astrocytic tumours (drawn online using MutationMapper, http://www.cbioportal.org/mutation_mapper.jsp). The relationship between mutation type, mutation site and domains of the *TP53* gene in primary astrocytic tumours (**a**) and recurrent astrocytic tumours (**b**). The sites with parentheses indicate those with a recurring mutation, and the number in brackets represents the mutation frequency of the site. AD (activation domain), DBD (DNA binding domain), TD (tetramerization domain).

In primary tumours, 21 (21/47, 44.68%) *IDH1* mutations were detected, all of which were the R132H mutation (CGT-CAT). In addition, 19 *TP53* mutations were found in 17 (36.17%, 17/47) primary samples (Fig. 1a); these mutations were missense or truncation mutations and involved 8 previously identified loci^30–35^: R175H (CGC-CAC), H193R (CAT-CGT), I195T (ATC-ACC), V216M (GTG-ATG), R273C (CGT-TGT), R273H (CGT-CAT), C275F (TGT-TTT) and R306 (CGA-TGA). Among the 17 samples in which *TP53* mutations were found, two contained two mutations each: R175H and R273C (No. 2) and I195T and R273C (No. 39). Fifteen (15/47, 31.91%) *TERTp* mutations were found in primary tumour tissue, including 12 cases of C228T (−124 bp) and 3 cases of C250T (−146 bp).

In recurrent tumours, all 22 (22/48, 45.83%) of the detected *IDH1* mutations were R132H (CGT-CAT). Eighteen *TP53* mutations were detected in 17 (35.42%, 17/48) recurrent tumours; again, these mutations were missense or truncation mutations. In addition, two other previously reported mutation sites were detected: P177R (CCC-CGC)^36^ and R273Y (CGT-TAT)^24^ (Fig. 1b). Furthermore, patient No. 39 lost the mutation at codon 273 H, and only the I195T mutation was found in the recurrent tumour. In recurrent tumour tissue, 16 (16/48, 33.33%) *TERTp* mutations were found, including 14 cases of C228T (−124 bp) and 2 cases of C250T (−146 bp).

### *IDH1*, *TP53* and *TERTp* mutation status and MGMT and ATRX protein expression levels were consistent in primary and recurrent gliomas

**Figure 2:**
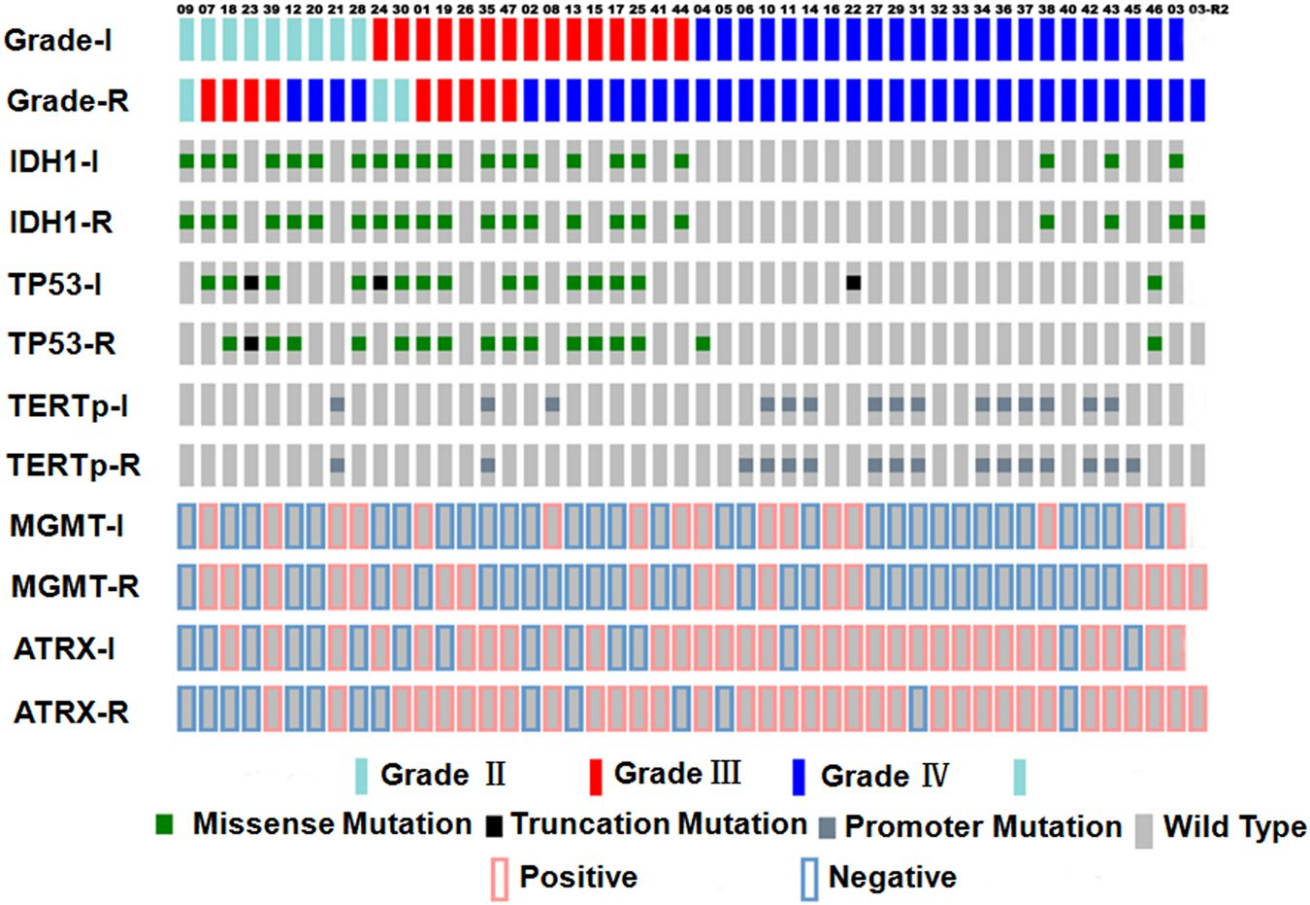
Mutation type in *IDH1*, *TP53* and *TERTp* and protein expression level of MGMT and ATRX in 47 paired cases of astrocytic tumours (drawn online using OncoPrinter, http://www.cbioportal.org/oncoprinter.jsp). P (primary astrocytic tumours), R (recurrent astrocytic tumours).

The *IDH1*, *TP53* and *TERTp* status in primary and corresponding recurrent tumours was analysed by McNemar’s test, and no significant differences were found (all p values were approximately equal to 1, Table 2). Furthermore, the consistency analysis results showed that all kappa values were quite high, and the recurrent tumours had consistent *IDH1*, *TP53* and *TERTp* status with their matched primary tumours (κ = 1.00, κ = 0.724, κ = 0.856; Table 2). All three investigated biomarkers were consistent in primary tumours and matched recurrences in 38 cases. The other 9 cases showed inconsistencies in the *TP53* or *TERTp* biomarker (Fig. 2). *TP53* status changed in 6 cases, among which 3 cases gained *TP53* mutations upon recurrence. In the other 3 cases, the *TP53* mutations detected in the primary tumours were not found in the recurrent tumours. *TERTp* status of the recurrent tumour was different from that of the primary tumour in 3 cases. One primary tumour was *TERTp* mutant, while the matched recurrent tumour was wild type. In contrast, the other 2 primary tumours were *TERTp* wild type, while *TERTp* mutations were found in the matched recurrent tumours (Fig. 2).

**Table 2:** *IDH1*, *TP53* and *TERT*p mutation status and MGMT and ATRX loss are stable in recurrent tumours compared with primary tumours.

			Recurrence										Measure of agreement		McNemar test
			*IDH1*		*TP53*		*TERTp*		MGMT		ATRX		Kappa	p	p
			MUT	WT	MUT	WT	MUT	WT	+	−	+	−			
Primary	*IDH1*	MUT	21	0									1	P < 0.001	p > 0.05
		WT	0	26											
	*TP53*	MUT			14	3							0.724	P < 0.001	p > 0.05
		WT			3	27									
	*TERTp*	MUT					14	1					0.856	P < 0.001	p > 0.05
		WT					2	30							
	MGMT	+							11	5			0.487	P = 0.001	p > 0.05
		−							6	25					
	ATRX	+									27	5	0.452	P = 0.002	p > 0.05
		−									6	9			

+ positive; − negative; WT wild type, MUT mutation.

The McNemar’s test did not show significant differences in ATRX and MGMT status between primary tumours and recurrences (all p values were approximately equal to 1, Table 2). The agreement test showed that ATRX and MGMT status in recurrent tumours and their primary counterparts was moderately consistent (κ = 0.452; κ = 0.487, Table 2). Neither ATRX nor MGMT protein expression changed in 31 cases of recurrent tumours. In the remaining 16 cases, 10 showed a change in ATRX or MGMT protein expression level, and 6 showed a change in both protein biomarkers (Fig. 2).

In all, the five studied biomarkers did not change in 25 cases upon recurrence (Fig. 2, Supplementary Table S1). The other 22 cases showed changes in one or two biomarkers upon comparison of recurrent tumours and primary tumours (Fig. 2, Supplementary Table S1). Clinicopathological features of primary and recurrent tumours and median PFS were not significantly different between the two groups (Supplementary Table S2).

### Prognostic value of *IDH1*, *TP53* and *TERTp* mutation and MGMT and ATRX expression level in astrocytic tumours

**Figure 3:**
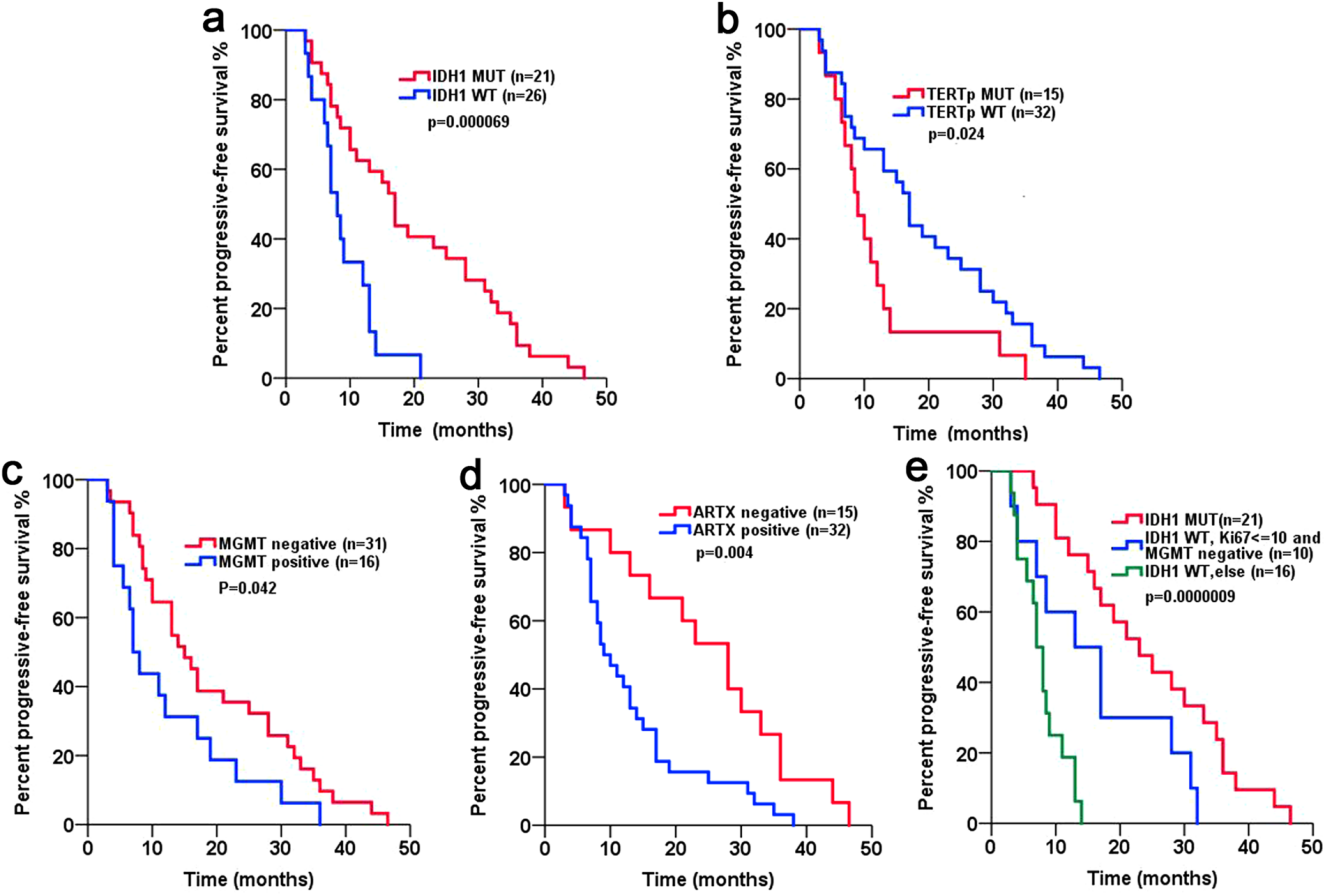
The relationships between molecular markers and progression-free survival (PFS). (**a**) Patients with IDH mutations showed a significantly improved PFS. (**b**) Patients with *TERTp* mutations showed a shorter PFS compared to those with *TERTp* wild type. (**c**) MGMT-positive patients had a shorter PFS compared to MGMT-negative patients. (**d**) ATRX-negative patients had a significantly improved PFS. (**e**) Among *IDH1* wild type patients, those with a low proliferative index and MGMT loss had a long PFS.

The value of *IDH1*, *TP53*, and *TERTp* mutations and MGMT and ATRX protein expression level in primary tumours for evaluating the PFS of patients with astrocytic tumours was analysed using the Kaplan-Meier survival estimator. PFS was significantly longer in patients harbouring *IDH1* mutations and in MGMT-negative or ATRX-negative patients (p = 0.000069, Fig. 3a; p = 0.042, Fig. 3c; p = 0.004, Fig. 3d; Table 3). In contrast, *TERTp* mutation was associated with a shorter PFS in patients with astrocytic tumours (p = 0.024, Fig. 3b; Table 3). Combined with clinical features and immunophenotype, a multivariate model for PFS was established (Table 3). In this model, *IDH1* mutation status, MGMT loss and low Ki67 index were significantly associated with a favourable influence on PFS (p = 0.04, p = 0.001, p = 0.009, Table 3). To further investigate the prognosis of the *IDH1*WT (*IDH1* wild type) group, we combined MGMT expression level and the Ki67 index to evaluate PFS. We classified 47 astrocytic tumours into 3 groups: Group 1, patients with *IDH1*MUT (*IDH1* mutant) regardless of Ki67 index or MGMT expression level; Group 2, patients with *IDH1*WT, low Ki67 index and MGMT protein loss; and Group 3, patients with *IDH1*WT but not classified as Group 2. Patients from Group 1 had the longest PFS, whereas patients from Group 3 had the shortest PFS. The PFS of Group 2 was between that of Groups 1 and 3. The median PFS of Groups 1, 2 and 3 was 23 months, 15 months and 7.5 months, respectively (p = 0.0000009, Fig. 3e). To investigate whether this new comprehensive classification is a better prognostic marker for PFS than the individual biomarkers (*IDH1*, Ki67 and MGMT), receiver-operating characteristic (ROC) curves of 1-year PFS were generated. The AUC (area under curve) of the comprehensive classifier for 1-year PFS was 0.792, which was greater than that for *IDH1* (0.732), Ki67 (0.618) or MGMT (0.623) alone in our dataset (Supplementary Table S3).

**Table 3:** Cox regression analysis of the progression-free survival of patients with astrocytic tumours.

variable	n	Median PFS	univariate analysis	multivariate analysis		
		(months)	p-value	RR	95% CI	p-value
Clinical features (primary)						
Age (>45 VS ≤ 45 years)			0.015	0.965	0.323–2.887	0.949
>45	29	10				
≤45 years	18	19				
Sex (Male VS Female)			0.841			
Male	31	13				
Female	16	12				
WHO grade (II/III vs IV)			0.000021	0.546	0.192–1.550	0.256
II/III	23	23				
IV	24	8.5				
Immunophenotype (primary)						
Ki67 (>10 VS ≤ 10)			0.000425	2.827	1.270–6.291	0.009
Ki67 > 10	14	8				
Ki67 ≤ 10	33	17				
MGMT (+ VS −)			0.042	3.761	1.770–7.992	0.001
+	16	7				
−	31	15				
ATRX (+ VS −)			0.004	0.553	0.242–1.266	0.161
+	32	9				
−	15	28				
Mutation statuses (primary)						
TP53 (MUT VS WT)			0.193			
MUT	17	21				
WT	30	9				
IDH1 (MUT VS WT)			0.000069	0.354	0.132–0.951	0.04
MUT	21	23				
WT	26	8				
*TERT* p (MUT VS WT)			0.024	1.279	0.590–2.771	0.534
MUT	15	17				
WT	32	9				

+ Positive, − Negative; MUT, mutation; WT, wild type.

The prognostic significance of *IDH1*, *TP53*, and *TERTp* mutations and loss of MGMT and ATRX in primary tumours for overall survival (OS) was evaluated using Kaplan-Meier analysis. *IDH1* and *TP53* mutations and loss of ATRX were associated with a better OS (p = 0.014, Supplementary Figure S1a; p = 0.016, Supplementary Figure S1c; p = 0.022, Supplementary Figure S1g). OS was significantly shorter in patients harbouring *TERTp* mutation (p = 0.001, Supplementary Figure S1e). There was no difference in OS between patients with or without MGMT loss. *IDH1*, *TP53* and *TERTp* mutation and ATRX loss at recurrence retained prognostic value for OS (p = 0.014, Supplementary Figure S1b; p = 0.005, Supplementary Figure S1d; p = 0.004, Supplementary Figure S1f; p = 0.03, Supplementary Figure S1h). Unfortunately, despite our best efforts, there were twenty-two patients lost to follow-up, which may lead to attrition bias. We did not do further analysis using Cox regression models in view of withdraw bias. Further study is required to confirm our observations.

## Discussion

With the exception of grade I astrocytic tumours, astrocytic tumours show diffuse infiltration of brain tissue and cannot be completely removed by surgical resection, which invariably leads to recurrent episodes. Recurrence remains one of the most important limitations in the cure of astrocytic tumours. Astrocytic tumours follow Darwin’s theory of evolution and acquire novel mutations, which account in part for treatment failure and recurrence^37^. *IDH1*, *TP53* and *TERTp* play key roles in tumourigenesis. However, few studies have investigated their mutation state in recurrent gliomas, particularly regarding *TERTp* mutation. In our study, similar to previously reported studies^22,38,39^, the *IDH1* status of the primary tumour was consistent with that of the corresponding recurrent tumour in all cases. However, previous studies reported that the *IDH1* mutation status changed in a minority of recurrent tumours^4,40^. The *TP53* status remained unchanged in 85.11% of the recurrent tumours in our group, which is close to the percentage reported by Groenendijk *et al*. (85.19%)^41^ and Kraus *et al*. (72.73%)^24^. To the best of our knowledge, the articles by Heidenreich *et al*.^42^ and Nonoguchi *et al*.^10^ are the only two describing *TERTp* mutations in adult malignant gliomas and matched recurrences. Two (2/20)^42^ and three (3/21)^10^ primary/recurrent tumour pairs with *TERTp* mutation in the primary lesion but not the recurrent lesion have been described. Our research confirmed that *TERTp* status changed in recurrent tumours in only a small percentage of cases (3/47). In all cases, *IDH1*, *TP53*, and *TERTp* status in the primary tumour always predicted the status in the recurrent tumour. These results suggest that alterations in *IDH1*, *TP53*, and *TERTp* occur early during gliomagenesis and remain stable in tumour recurrence.

*ATRX* mutation leads to a truncated protein and is highly associated with negative staining by immunohistochemistry, which can be used as an alternative indicator for ATRX mutation^43^. In contrast to the pattern of *TERTp* mutations, which were predominately detected in pGBM, ATRX protein loss was mostly detected in A, AA and sGBM. These results suggest that astrocytomas and pGBM maintain telomere length while cells divide by altering different genes. In our samples, 11 of 47 cases (23.40%) showed a change in ATRX expression status, which was in agreement with the work by Johnson *et al*.^38^.

MGMT greatly improves tumour cell resistance to alkylating nitrosoureas and methylating agents by repairing alkylating lesions in DNA. Loss of MGMT expression in glioma is rarely caused by deletion, mutation, or rearrangement of the *MGMT* gene, but is mainly caused by methylation of the upstream promoter^44^. A significant inverse correlation was observed between MGMT protein expression and MGMT promoter methylation^44–46^. Notably, regulation of MGMT expression is a complicated process in which promoter hypermethylation is not the sole determinant^27^. Thus, an inconsistent correlation between abnormal promoter methylation and protein expression loss has been observed in some studies^47,48^. To a certain extent, the limitations of promoter methylation and protein expression detection methods interfere with the evaluation of correlations. Although promoter methylation can be directly and objectively detected, the methods for analysing *MGMT* promoter methylation status are more complicated than those for detecting MGMT protein expression by immunohistochemistry, which is a technique that is available in most laboratories. In our study, MGMT protein expression status by immunohistochemistry was changed in 11 of 47 cases (23.40%) during recurrence. The probability of change in negative tumours was equal to that in positive tumours; this observation is different from that reported by Brandes *et al*.^49^. Previous studies reported that MGMT methylation status changed after recurrence in 10% to 58.3% of the patients^19,41,49,50^. In brief, MGMT and ATRX protein expression levels were stable during recurrence in most cases.

All five biomarkers (*IDH1*, *TP53* and *TERTp* mutation status and MGMT and ATRX protein expression levels) remained unchanged in 25 cases during recurrence, suggesting that these recurrences might represent direct expansion of the primary residual tumour and may stem from linear clonal evolution^38^. Another possibility is that these alterations occurred early in tumour development, prior to branched clonal evolution. The other 22 cases showed diversity in one or two biomarkers. Among these 22 cases, some acquired novel gene mutation or protein loss during recurrence, perhaps following Darwin’s theory of evolution. The other cases in which gene mutation or protein loss detected in the primary tumour was no longer found in the recurrent tumour could be partly explained by considering that the recurrent tumours in these patients were seeded by cells derived from the primary tumour at an early stage of evolution^39^. The genotype changes during recurrence and may follow the pattern of branched clonal evolution^38^.

To a certain extent, the histological and clinical features of astrocytic tumours can be prognostic indicators. However, to date, it is unclear why the interval between first surgery and recurrence is long in some patients but relatively short in others. Although Thon *et al*.^21^ reported that patients with *IDH1* mutation grade II A had a short PFS, other studies have shown that A and AA^22^ and pGBM^11^ patients with *IDH1* mutations have a significantly longer PFS. Our data also showed that patients with astrocytic tumours harbouring *IDH1* mutations had a significantly longer PFS than those with wildtype *IDH1*. Furthermore, Hartmann *et al*.^11^ found that patients with *IDH1*-mutant glioblastoma have a more favourable PFS than those with *IDH1-*WT AA. All together, these observations indicate that *IDH1* mutation can be considered a prognostic marker of PFS in patients with astrocytic tumours. Together with *IDH1* gene mutation, MGMT protein expression level and Ki67 index were identified as independent prognostic factors for PFS in multivariate Cox regression analysis. The value of Ki67 and MGMT as independent prognostic factors in glioma was reported previously^51–55^. We classified 47 astrocytic tumours into 3 groups based on *IDH1* mutation status and MGMT and Ki67 expression level without considering histological grade. Group 1 patients (*IDH1*MUT regardless of Ki67 index or MGMT expression level) had the longest PFS, whereas Group 3 patients (*IDH1*WT but not in Group 2) had the shortest PFS. Group 2 patients (*IDH1*WT, low Ki67 index and MGMT protein loss) had an intermediate PFS between that of Groups 1 and 3. The AUC of the comprehensive classifier at the first year was greater than *IDH1*, Ki67 or MGMT alone in our dataset, suggesting that *IDH1* mutation combined with MGMT protein expression level and Ki67 index is a better prognostic factor for PFS in patients with astrocytic tumours. Therefore, we propose this new molecular classification for evaluating the PFS of patients with astrocytic tumours in future clinical trials. We focused on determining the prognostic significance of certain markers. In the future, further clinical studies are needed to clarify whether these markers are predictive for treatment response^56^.

In conclusion, our study reveals that in most cases, *IDH1*, *TP53* and *TERTp* mutation status and MGMT and ATRX expression levels are stable during disease recurrence, perhaps indicating that those investigated biomarker alterations occurred early in the astrocytic tumour development. Meanwhile, in *IDH1*WT group, patients who were negative for MGMT and a low Ki67 index had a longer PFS than those who were positive for MGMT or had a high Ki67 index. The combination of *IDH1* mutation, MGMT protein expression level and Ki67 index is a better indicator of the survival of patients with astrocytic tumours than any of these indicators alone. Therefore, *IDH1* mutation combined with MGMT protein expression level and Ki67 index is a potential novel biomarker for evaluating the PFS of patients with astrocytic tumours. A larger study is required to confirm our observations.

## Methods

### Patients

Forty-seven paired astrocytic tumour samples were obtained from Xijing Hospital. Forty-six patients had a primary tumour and one recurrent tumour, and one patient had a primary tumour and two subsequent recurrent tumours. Tumour specimens were obtained by surgical resection between January 2006 and March 2015. Pathological diagnosis was reconfirmed by two neuropathologists in the Department of Pathology of Xijing Hospital under the 2007 WHO Classification of Tumours of the Central Nervous System. All primary tumours were untreated prior to operation and underwent total gross resection. The clinical data for each patient are shown in Table 4. All the study protocols were approved by the ethics committee of Xijing Hospital, Fourth Military Medical University. All study procedures were performed in accordance with the approved guidelines and regulations of the Fourth Military Medical University. Informed consent was obtained from all the patients, and all patient records were anonymised according to ethical and legal standards.

**Table 4:** Patient clinical data and pathological diagnosis of primary tumours and recurrences.

Patient	Gender	Age^a^ (years)	Diagnosis^b^ prim	Interval^c^ (months)	Diagnosis^d^ 1st rec	Interval^e^ (months)	Diagnosis^f^ 2nd rec	Adjuvant therapy^g^ prim	Adjuvant therapy^h^ 1st rec
1	M	41	AA	7	sGBM			RT	CH
2	F	51	AA	36	AA			CH	CH + RT
3	F	55	pGBM	6.5	rGBM1	12.5	rGBM2	CH + RT	CH
4	M	50	pGBM	8	rGBM1			CH + RT	CH + RT
5	M	61	pGBM	13	rGBM1			CH + RT	CH
6	M	56	pGBM	8.5	rGBM1			CH + RT	CH + RT
7	F	32	A	36	AA			None	CH + RT
8	M	28	AA	7	sGBM			RT	CH + RT
9	M	40	A	33	A			RT	CH + RT
10	F	46	pGBM	11	rGBM1			CH + RT	CH
11	M	65	pGBM	4	rGBM1			CH + RT	CH + RT
12	M	32	A	46.5	sGBM			RT	CH
13	M	49	AA	28	sGBM			RT	CH + RT
14	M	48	pGBM	13	rGBM1			CH + RT	CH + RT
15	F	59	AA	7	sGBM			CH	CH + RT
16	M	55	pGBM	4	rGBM1			CH + RT	CH
17	M	30	AA	10	sGBM			RT	CH + RT
18	M	26	A	25	AA			RT	CH + RT
19	M	39	AA	16	AA			CH	CH + RT
20	M	28	A	44	sGBM			None	CH + RT
21	F	53	A	5.5	sGBM			None	CH
22	F	54	pGBM	4	rGBM1			CH + RT	CH + RT
23	M	54	A	28	AA			None	CH + RT
24	F	31	AA	38	A			CH	CH + RT
25	F	21	AA	23	sGBM			CH + RT	CH
26	M	51	AA	32	AA			RT	CH + RT
27	M	49	pGBM	3	rGBM1			CH + RT	CH
28	M	32	pGBM	6	rGBM1			CH + RT	CH + RT
29	F	65	pGBM	8.5	rGBM1			CH + RT	CH + RT
30	F	38	AA	21	A			CH	CH + RT
31	M	76	pGBM	6.5	rGBM1			RT	CH
32	M	43	pGBM	7	rGBM1			CH + RT	CH + RT
33	F	54	pGBM	17	rGBM1			CH + RT	CH + RT
34	F	64	pGBM	8	rGBM1			CH + RT	CH + RT
35	M	46	AA	35	AA			CH	CH + RT
36	M	58	pGBM	9	rGBM1			CH + RT	CH
37	M	50	pGBM	14	rGBM1			CH + RT	CH + RT
38	M	63	pGBM	12	rGBM1			CH + RT	CH + RT
39	F	23	A	17	AA			RT	CH + RT
40	M	57	pGBM	13	rGBM1			CH + RT	CH
41	M	14	AA	3.5	sGBM			CH + RT	CH + RT
42	M	59	pGBM	31	rGBM1			CH + RT	CH + RT
43	F	60	pGBM	10	rGBM1			CH + RT	CH + RT
44	M	40	AA	19	sGBM			CH	CH + RT
45	M	53	pGBM	3	rGBM1			CH + RT	CH
46	F	54	pGBM	17	rGBM1			CH + RT	CH + RT
47	M	27	AA	15	AA			CH	CH + RT

^a^Age at first operation. ^b^Prim, primary; A, astrocytoma; AA, anaplastic astrocytoma; pGBM, primary glioblastoma. ^c^Interval between the first and second operations. ^d^1st rec, first recurrence; sGBM, secondary glioblastoma; rGBM_1_, the first recurrence of primary glioblastoma. ^e^Interval between the second and third operations. ^f^2nd rec, second recurrence; rGBM_2_, the second recurrence of primary glioblastoma; ^g^ ^h^RT, radiotherapy; CH chemotherapy.

### Molecular Genetic Analysis

Whole resected tumour tissues from individual patients were sampled to extract tumour DNA. Genomic DNA was extracted from formalin-fixed, paraffin-embedded tissues using the Qiagen GeneRead^TM^ FFPE DNA Extraction Kit. Before the tumour DNA was isolated, the proportion of tumour in the consecutive haematoxylin and eosin-stained section was evaluated. If the proportion of tumour was more than ninety percent, five 10-μm-thick tissue sections were placed directly in a sterile 1.5-ml Eppendorf tube for DNA extraction. If the proportion of tumour was less than ninety percent, tumour-containing regions were removed from micro-dissected 10-μm sections to avoid contamination with normal tissue. After the tumour DNA was isolated, DNA quantity and quality were analysed using a Nanodrop 2000 spectrophotometer and 0.5% agarose gels.

### Detection of *TERTp*, *TP53* and *IDH1* Hotshot Mutations

Mutations were analysed by PCR and direct sequencing. Primer sequences and amplification conditions for exons 5–8 of *TP53* and *IDH1* codon 132 mutations were previously reported^57,58^. Primer sequences for *TERTp* were designed using the Primer Premier software package (version 5, Premier Inc.). Primer sequences and amplification conditions for *IDH1*, *TP53* and *TERTp* are shown in detail in Supplementary Table S4.

### Immunohistochemistry analysis

Immunohistochemistry was performed to detect the protein expression of MGMT and ATRX in the tissue samples. Briefly, 3-µm-thick tissue sections were prepared from formalin-fixed, paraffin-embedded tissue. Then, sections were deparaffinised in xylene and rehydrated in a series of ethanol washes. The sections were treated with 0.01 M sodium citrate buffer (pH 6.0) for 1 minute using a steam pressure cooker for antigen retrieval. The sections were immersed in 3% H_2_O_2_ for 15 minutes at room temperature to block endogenous peroxidase activity. After washing with phosphate-buffered saline (PBS) three times, sections were blocked in 5% bovine serum albumin (BSA) for 30 minutes. The sections were incubated at 4 °C overnight with anti-ATRX antibody (cat# HPA001906, lot J104918, Sigma-Aldrich, St. Louis, USA; dilution 1:500) or anti-MGMT antibody (clone MT3.1, MAB-0361, MAIXIN, Fuzhou, China; dilution 1:100). The next day, the sections were washed with PBS three times and then incubated with secondary antibody (REAL^TM^ EnVision^TM^ HRP anti-rabbit/mouse K5007, lot 20015510, Dako, USA) at room temperature for 30 minutes. The positive signal was visualised using diaminobenzidine as the chromogen, and nuclei were counterstained with haematoxylin. Blood vessel endothelium was used as an internal positive control, and PBS was used as a negative control. The immunoreactivity to ATRX and MGMT protein was evaluated by estimating the proportion of positive cells: ≤ 10% was regarded as negative, and > 10% was regarded as positive^59,60^. The stained sections were scored by two pathologists. Discrepancies between the two pathologists were settled by additional observation of the specimens and discussion until consensus was achieved. The pathologists were blinded to the clinical and molecular information during the immunohistochemical analysis.

### Statistical Analysis

The agreement and difference in *IDH1*, *TP53* and *TERTp* mutation status and MGMT and ATRX expression level in primary and recurrent tumours were estimated using the agreement test and McNemar’s test, respectively. The relation between molecular markers and clinical data was analysed using the chi-square test. PFS was defined as the interval between first surgical treatment and tumour recurrence. The PFS of different groups was estimated by Kaplan-Meier analysis, and the prognostic difference was evaluated with the log-rank test. Then, multivariate Cox regression models were used to evaluate potential prognostic factors. The new comprehensive classification was validated using ROC curves. P values were all two-tailed, and p values less than 0.05 indicated statistical significance. All data were analysed using SPSS software package (version 23, SPSS Inc.).

### Data Availability

All data generated or analysed during this study are included in this published article (and its Supplementary Information files).

## Electronic supplementary material

Primary Astrocytic Tumours and Paired Recurrences have Similar Biological Features in IDH1, TP53 and TERTp Mutation and MGMT, ATRX Loss

Datset 1
