# Supplementary material for: Primary Astrocytic Tumours and Paired Recurrences have Similar Biological Features in IDH1, TP53 and TERTp Mutation and MGMT, ATRX Loss
Source: Sci Rep. 2017 Oct 12;7:13038. doi: 10.1038/s41598-017-13272-9 (PMC5638900; doi:10.1038/s41598-017-13272-9)
Supplement: Supplementary file 1 — Primary Astrocytic Tumours and Paired Recurrences have Similar Biological Features in IDH1, TP53 and TERTp Mutation and MGMT, ATRX Loss [file 41598_2017_13272_MOESM1_ESM.doc]

**Primary Astrocytic Tumours and Paired Recurrences have Similar Biological Features in IDH1, TP53 and TERTp Mutation and MGMT, ATRX Loss**

**Xia Li, Jie Wei, Yixiong Liu, Peifeng Li, Linni Fan, Yingmei Wang, Mingyang Li, Danhui Zhao, Zhou Yu, Jing Ye, Ying Guo, Qingguo Yan, Shuangping Guo, Zhe Wang** *

State Key Laboratory of Cancer Biology, Department of Pathology, Xijing Hospital and School of Basic Medicine, The Fourth Military Medical University, Xi'an, Shaan Xi Province, China.

*corresponding author: zhwang@fmmu.edu.cn

**Supplementary Table**

**Supplementary Table S2**. Relationship between biomarkers statuses and clinicopathological features of 47 patients with astrocytic tumour.

|  | | five biomarkers statuses | |  |
| --- | --- | --- | --- | --- |
| variable | | Changed cases | Unchanged cases | p-value |
| Clinical features | | | | |
| Age | | | | |
| primary | >45 years | 11 | 18 | 0.122 |
|  | ≤45 years | 11 | 7 |  |
| recurrent | >45 years | 11 | 18 | 0.122 |
|  | ≤45 years | 11 | 7 |  |
| Sex | |  |  |  |
|  | Male | 14 | 16 | 0.85 |
|  | Female | 7 | 9 |  |
| WHO grade | | | | |
| primary | Ⅱ/Ⅲ | 9 | 14 | 0.302 |
|  | Ⅳ | 13 | 11 |  |
| recurrent | Ⅱ/Ⅲ | 8 | 4 | 0.11 |
|  | Ⅳ | 14 | 21 |  |
| Immunophenotype | | | | |
| Ki67 index | | | | |
| primary | Ki67>10 | 5 | 9 | 0.321 |
|  | Ki67 ≤10 | 17 | 16 |  |
| recurrent | Ki67>10 | 9 | 16 | 0.113 |
|  | Ki67 ≤10 | 13 | 9 |  |

**Supplementary Table S3**. Areas under the curve (AUC) values of *IDH1* mutations, Ki67 index, *MGMT* protein level and the comprehensive predictor retrieved from ROC analyses at one-year PFS.

|  | | 1-year PFS | |  |
| --- | --- | --- | --- | --- |
| variable | | Recurrent cases | Unrecurrent cases | AUC |
| *DH1* | |  |  | 0.732 |
|  | MUT | 4 | 17 |  |
|  | WT | 17 | 9 |  |
| MGMT | |  |  | 0.623 |
|  | Positive | 10 | 6 |  |
|  | Negative | 11 | 20 |  |
| Ki67 index |  |  |  | 0.618 |
|  | Ki67>10 | 9 | 5 |  |
|  | Ki67≤10 | 12 | 21 |  |
| Comprehensive predictor |  |  |  | 0.792 |
|  | Group 1 | 4 | 17 |  |
|  | Group 2 | 4 | 6 |  |
|  | Group 3 | 13 | 3 |  |

WT (wild type), MUT (mutation type)

**Supplementary Table S4**. Primer sequences and amplification conditions of *IDH1*, *TP53* and *TERT* promoter

| Gene | Primer sequence | condition | | | | Product  size(bp) |
| --- | --- | --- | --- | --- | --- | --- |
|  |  | denaturation | anneal | extension | cycle |  |
| IDH1 | F：CGGTCTTCAGAGAAGCCATT | 95℃, 30s | 56℃, 40s | 72℃, 50s | 35 | 129 |
|  | R：GCAAAATCACATTATTGCCAAC |  |  |  |  |  |
| TP53-5 | F：TGCCCTGACTTTCAACTCTG | 94℃, 40s | 55℃, 40s | 72℃, 60s | 35 | 233 |
|  | R：GCTGCTCACCAT CGCTAT |  |  |  |  |  |
| TP53-6 | F：CTGATTCCTCACTGATTGCT | 94℃, 40s | 55℃, 40s | 72℃, 60s | 35 | 153 |
|  | R：AGTTGCAAACCAGACCTCAGG |  |  |  |  |  |
| TP53-7 | F：CCTGTGTTATCTCCTAGGTTG | 94℃, 40s | 55℃, 40s | 72℃, 60s | 35 | 169 |
|  | R：GCACAGCAGGCCAGTGTGCA |  |  |  |  |  |
| TP53-8 | F：GACCTGATTTCCTTACTGCC | 94℃, 40s | 55℃, 40s | 72℃, 60s | 35 | 219 |
|  | R：TCTCCTCCACCGCTTCTTGT |  |  |  |  |  |
| TERT promote | F：TCCTGCCCCTTCACCTTCCA | 94℃, 30s | 60℃, 30s | 72℃, 120s | 30 | 251 |
|  | R：GCAGCAGGGAGCGCACGG |  |  |  |  |  |


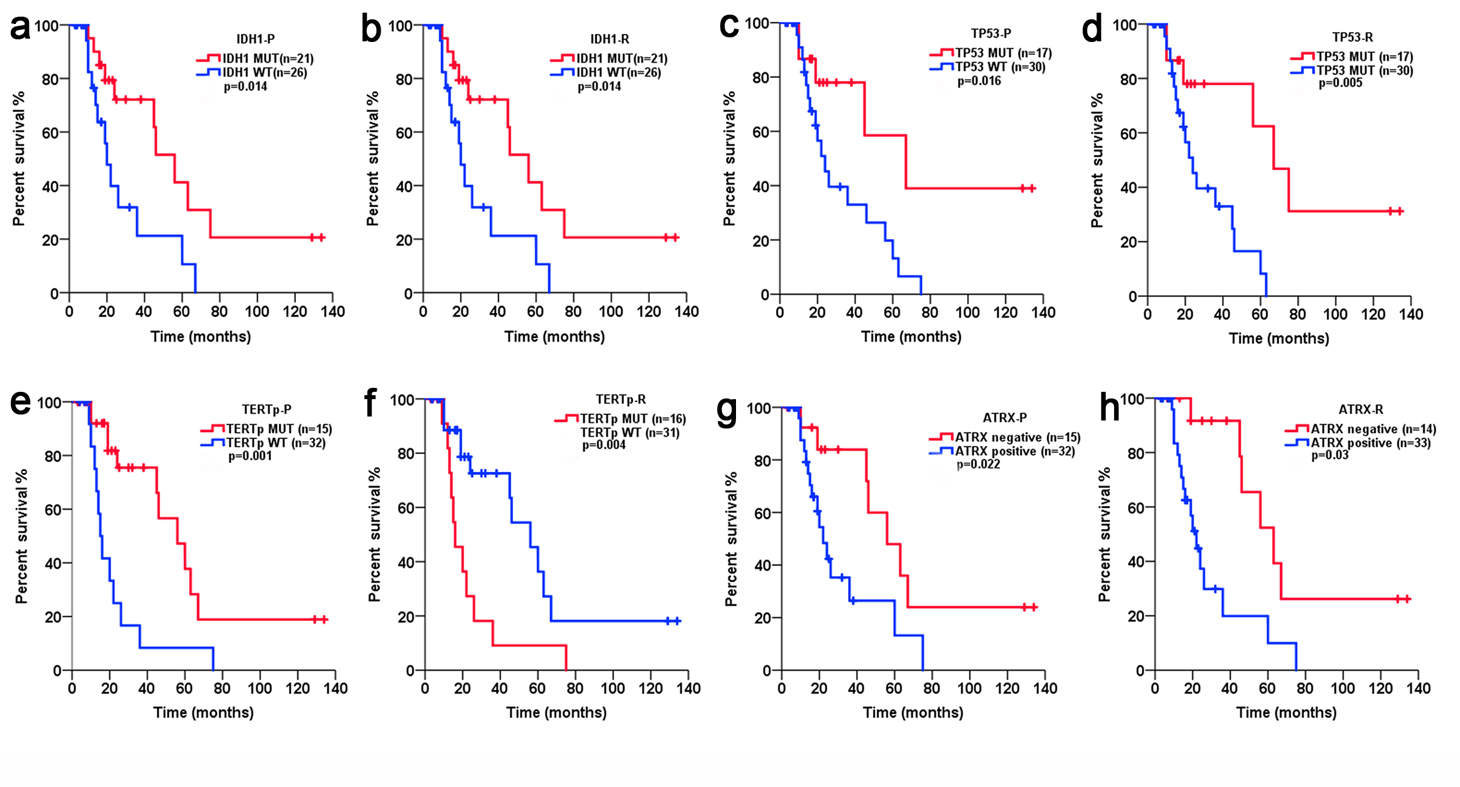


**Supplementary Figure 1**. The relationships between molecular markers and overall survival (OS). Patients with IDH mutations showed a significantly improved OS; *IDH1* mutations in primary tumours a), *IDH1* mutations in recurrence b). Patients with *TP53* mutations showed a longer OS compared to those with *TP53* wild type; *TP53* mutations in primary tumours c), *TP53* mutations in recurrence d). Patients with *TERTp* mutations showed a shorter OS compared to those with *TERTp* wild type; *TERTp* mutations in primary tumours e), *TERTp* mutations in recurrence f). ATRX-negative patients had a significantly improved OS; ATRX expression in primary tumours g), ATRX expression in recurrence h). P (primary astrocytic tumours), R (recurrent astrocytic tumours).
